# Textbook outcome in surgery for primary hyperparathyroidism: first steps toward a composite quality metric

**DOI:** 10.1007/s13304-026-02573-2

**Published:** 2026-05-28

**Authors:** Juan José Ruiz-Manzanera, Beatriz Febrero, Natalia Escavy, Miriam Abellán, María Teresa Soriano, Alfonso Aliaga, Inmaculada Ros-Madrid, José M. Rodríguez

**Affiliations:** 1 Endocrine Surgery Unit, General and Digestive Surgery, Virgen de la Arrixaca University Clinical Hospital, Murcia, Spain; 2 Murcian Institute of Biosanitary Research Pascual Parrilla (IMIB Pascual Parrilla), Murcia, Spain; 3 Department of Surgery, Pediatrics, Obstetrics and Gynecology. University ofMurcia (UMU), Murcia, Spain; 4 Endocrinology Service, Virgen de la Arrixaca University Clinical Hospital, Murcia, Spain

**Keywords:** Primary hyperparathyroidism, Parathyroidectomy, Textbook outcome, Quality indicators, Surgical education

## Abstract

Textbook Outcome (TO) is a composite quality metric reflecting the ideal postoperative course through the simultaneous achievement of multiple desirable endpoints. Although widely explored in other surgical specialties, its application in endocrine surgery remains limited, and has not been specifically assessed in primary parathyroidectomy for sporadic primary hyperparathyroidism. A retrospective cohort study was conducted including 100 consecutive patients who underwent parathyroidectomy for sporadic primary hyperparathyroidism (PHPT) between January 2019 and February 2023 at a tertiary endocrine surgery unit. TO was defined by the absence of postoperative complications (wound events, symptomatic hypocalcemia, vocal cord dysfunction), hospital stay ≤ 24 h, histopathological confirmation of parathyroid tissue, absence of 90-day readmission or mortality, biochemical cure at six months, and absence of recurrence at one year. Descriptive and univariate analyses were performed. TO was achieved in 78% of patients. The most frequent reasons for failure were hospital stay longer than 24 h (n = 7) and lack of biochemical cure at six months (n = 7). Most failures (68.2%) were due to a single unmet criterion. In univariate analysis, absence of dyslipidemia (*p* = 0.016), single-gland resection (*p* = 0.022), and histopathological confirmation of a solitary adenoma (*p* = 0.026) were significantly associated with TO achievement. Application of TO to parathyroidectomy for sporadic PHPT proved feasible and informative. The analysis of failures identified prolonged hospitalization and lack of biochemical cure at six months as the most frequent reasons for non-achievement. TO may provide a comprehensive benchmark that complements traditional outcome measures and supports future quality improvement initiatives in endocrine surgery.

## Introduction

Primary hyperparathyroidism (PHPT) is a common endocrine disorder characterized by the autonomous overproduction of parathyroid hormone (PTH), typically resulting in hypercalcemia and a wide range of systemic manifestations [1, 2]. While its classical presentation includes nephrolithiasis, bone disease, and overt symptoms of hypercalcemia, most cases are currently diagnosed incidentally through routine biochemical testing, often in asymptomatic or oligosymptomatic individuals [3].

Parathyroidectomy remains the only curative treatment for PHPT, yielding excellent outcomes in terms of biochemical normalization and symptom improvement [3, 4]. As clinical presentations evolve and surgical techniques advance, there is an increasing need for comprehensive and standardized tools to evaluate surgical success.

In this context, the concept of Textbook Outcome (TO) has emerged as a composite quality metric in various surgical fields [5–7]. It is based on the simultaneous achievement of multiple desirable outcomes—such as absence of complications, short hospital stay, and sustained disease control—and provides a global snapshot of the ideal postoperative course. However, its application within endocrine surgery remains limited. One recent multicenter study by Nomine-Criqui et al. [8] applied a TO definition to reoperations for persistent or recurrent PHPT. However, no study to date has evaluated TO specifically in first-time, sporadic parathyroidectomy, nor adapted the TO framework to the perioperative profile and biochemical success parameters characteristic of primary PHPT.

Quality in endocrine surgery should not be assessed solely through isolated outcome measures, but also through composite indicators that better capture the complexity of real-world surgical care [5, 6]. Unlike single indicators such as biochemical cure, complication rate, or postoperative hypocalcemia—which are often uniformly high or low in PHPT and therefore provide limited differentiation—TO integrates multiple domains simultaneously. This composite structure allows TO to detect variations in perioperative performance, identify contributors to suboptimal recovery, and offer a more stringent benchmark. In the context of PHPT, where surgical outcomes are typically favorable, TO provides superior discriminatory value by capturing nuances in patient experience, resource utilization, and sustained disease control beyond what isolated metrics can convey.

TO may serve as a valuable benchmark to identify candidates for outpatient surgery, compare performance across surgeons and institutions, pinpoint areas for quality improvement, and enhance patient-centered outcomes [9]. Given the already high cure rates and low complication rates typically reported in PHPT surgery, single metrics may no longer be sufficiently discriminative. In contrast, composite indicators such as TO offer a more stringent and informative standard, especially in low-morbidity procedures.

The aim of this study was to assess the rate of achieving a TO after parathyroidectomy for sporadic PHPT. Secondary objectives included identifying the most frequent causes of TO failure and evaluating the association between patient variables and the likelihood of achieving TO.

## Methods

### Study design

A retrospective observational cohort study was carried out at a tertiary referral hospital. The study included all consecutive patients who underwent parathyroidectomy for sporadic PHPT between January 2019 and February 2023. Clinical, biochemical, surgical, and follow-up data were collected from electronic medical records and prospectively maintained surgical databases. The study was approved by the Ethical Committee of the Virgen de la Arrixaca University Clinical Hospital (Murcia, Spain).

### Study population

The patients were diagnosed with PHPT in the year preceding the study and surgical treatment was indicated. For PHPT diagnosis, values of PTH > 65 pg/ml [15–65] and calcium levels > 10.2 mg/dl [8.8–10.2] were taken into account. Patients with normocalcemic PHPT who experienced increased PTH (after ruling out other secondary causes) were also included. All the patients had normal levels of 25-hydroxyvitamin D. In relation to the terms, symptomatic PHPT is defined as patients with classic symptoms, taking into account both nephrourological and/or bone symptoms. Asymptomatic PHPT cases are defined as patients with a diagnosis of PHPT based on analytical abnormalities in the absence of classic symptoms [10, 11]. The indication for parathyroidectomy was determined by the appearance of symptoms in patients with PHPT. Alternatively, in relation to the cases of asymptomatic PHPT, the indication for surgical treatment was determined by the criteria established in the latest guidelines elaborated by the National Institutes of Health for parathyroidectomy [12, 13], in which the following criteria are included: serum calcium > 1.0 mg/dl above normal values; < 50 years; T-score < − 2.5 in lumbar spine, femoral neck or distal third of the radius; vertebral fracture evidenced by imaging test; creatinine clearance < 60 cc/min; calcium in 24-h urine > 400 mg/24 h and an increased risk of lithiasis by biochemical analysis; nephrolithiasis or nephrocalcinosis demonstrated by imaging tests.

All patients in the study received preoperative imaging studies using at least ultrasound and 99mTc-MIBI scintigraphy. A selective surgical approach was performed if uniglandular disease was identified on one or both preoperative imaging studies. The intraoperative parathyroid hormone method was used in patients to identify parathyroid adenoma. According to Miami criteria, the successful excision of parathyroid was considered a drop in PTH levels of > 50% at 10 min post excision from baseline [14].

Biochemical cure criteria were phenotype-specific. In hypercalcemic PHPT, cure was defined as restoration of normal calcium homeostasis maintained for at least 6 months. In normocalcemic PHPT, cure was defined as maintained normocalcemia with normal PTH levels at ≥ 6 months postoperatively. Patients with persistently elevated PTH were evaluated for secondary causes of hyperparathyroidism and, if none were identified, were monitored for recurrent or persistent disease [10, 15]. Follow-up was carried out one month after surgery, at 3 months, 6 months and one year after surgery. Recurrence was defined as the reappearance of hypercalcemia after an initial period of biochemical cure.

### Inclusion criteria

Patients diagnosed with sporadic PHPT who were indicated for surgical treatment and had at least one year of postoperative follow-up were included in the study.

### Exclusion criteria

Patients were excluded if they had hereditary forms of primary hyperparathyroidism, secondary or tertiary hyperparathyroidism. Patients were excluded if they had familial hypocalciuric hypercalcemia, determined using biochemical criteria including 24-h urinary calcium excretion and the calcium-to-creatinine clearance ratio. When a 24-h urine collection was not available, a spot Ca/Cr clearance ratio was used [16]. Patients undergoing reoperation for recurrent or persistent disease or those surgically treated for parathyroid conditions other than sporadic PHPT were also excluded.

### Definition of textbook outcome

The TO framework applied in this study was conceptually based on previously established composite outcome models developed in other surgical fields—most notably colorectal and hepatopancreatobiliary surgery—and then specifically adapted by the authors to the clinical characteristics and outcome profile of sporadic PHPT. Accordingly, the selected criteria were chosen to reflect domains that are consistently considered benchmarks of safe and effective parathyroid surgery. TO was defined as the achievement of an optimal postoperative course, based on the simultaneous fulfillment of the following nine criteria:

1. Absence of surgical wound complications (including seroma, wound infection, or wound suture dehiscence).
2. Absence of symptoms of hypocalcemia (such as paresthesia or tetany).
3. Absence of postoperative vocal cord dysfunction or documented injury to the recurrent laryngeal nerve.
4. Hospital stay ≤  24 h.
5. Histopathological confirmation of abnormal parathyroid tissue (adenoma, hyperplasia, or enlarged gland).
6. Absence of hospital readmission within 90 days.
7. Absence of mortality within 90 days.
8. Achievement of biochemical cure.
9. Absence of persistent or recurrent disease after parathyroidectomy.

Biochemical cure, as part of the TO composite, was assessed at 6 months according to preoperative biochemical phenotype. In hypercalcemic PHPT, cure was defined as normalization of serum calcium levels. In normocalcemic PHPT, cure was defined as maintained normocalcemia with normal PTH levels at follow-up, after exclusion of secondary causes of hyperparathyroidism. Absence of persistence or recurrence referred to the maintenance of normocalcemia at one year of follow-up. Although related, these two outcomes capture different time points and were deliberately included as separate criteria to reflect both early and mid-term surgical success. Histopathological confirmation of parathyroid tissue was included for completeness, acknowledging that this parameter is usually fulfilled and therefore has limited discriminatory power.

### Variables collected

The following variables were collected for each patient:

- Preoperative variables: age, sex, body mass index (BMI), comorbidities (hypertension, diabetes mellitus, dyslipidemia, chronic kidney disease, cardiovascular or pulmonary disease and thyroid disease), ASA classification, symptoms associated with PHPT (nephrolithiasis, osteoarticular pain, and nonspecific symptoms, including fatigue, cognitive complaints, muscle weakness, mood changes, and gastrointestinal discomfort) [17, 18], biochemical values (serum calcium, PTH and 25-hydroxyvitamin D), and imaging findings. Preoperative laryngeal assessment by flexible laryngoscopy was systematically performed when preoperative imaging failed to localize the gland or when bilateral cervical exploration was anticipated. Laryngoscopy was also carried out in patients with pre-existing dysphonia or other symptoms suggestive of recurrent laryngeal nerve dysfunction.
- Intraoperative variables: type of surgical approach (focused, bilateral, or unilateral exploration), number of glands resected, intraoperative PTH response, and intraoperative complications.
- Postoperative variables: length of hospital stay, complications related to the surgical wound, presence of symptoms of hypocalcemia or dysphonia, mortality and readmission within 90 days, histopathological findings, serum calcium and PTH at 6 and 12 months, and recurrence/persistence. Postoperative dysphonia was defined as patient-reported voice changes confirmed by clinical assessment. Laryngoscopy was performed in cases with persistent symptoms beyond the immediate postoperative period. Intraoperative nerve monitoring was routinely used in all cases.

In our institution, parathyroidectomy is performed under routine inpatient admission. Patients spend the immediate postoperative period in the recovery unit before overnight ward stay. Serum calcium is systematically checked at 24 h, and discharge is generally planned on the morning of postoperative day 1 if clinically stable. Therefore, hospital stay beyond 24 h reflects a deviation from expected recovery and was included as a clinically meaningful TO criterion. This length-of-stay benchmark reflects institutional postoperative pathways and may vary according to reimbursement models or healthcare systems; accordingly, its interpretation should be contextualized within this single-center framework.

### Statistical analysis

Descriptive statistics were performed for all variables. Categorical variables were summarized as frequencies and percentages, while continuous variables were expressed as mean and standard deviation or median and interquartile range (IQR), depending on data distribution. An initial descriptive analysis was carried out for the entire cohort. For the purposes of univariate comparisons, cervical exploration was dichotomized as focused vs. non-focused (including unilateral and bilateral exploration). BMI was categorized as ≥ 25 vs. < 25, and ASA classification as I–II vs. ≥ III. Odds ratios and 95% confidence intervals were calculated for categorical variables. To evaluate factors associated with achieving the TO, univariate analyses were performed. TO was considered as the dependent binary variable. Patient variables were included in this analysis. For continuous variables, Student’s t-test was used, preceded by Levene’s test to assess the equality of variances. For categorical variables, Pearson’s chi-squared test or Fisher’s exact test was applied, as appropriate. Statistical significance was set at a *p*-value < 0.05. All analyses were performed using *IBM SPSS Statistics version 30.0* (*IBM Corp., Armonk, NY, USA*).

## Results

### Baseline characteristics of the study population

A total of 100 patients who underwent parathyroidectomy for sporadic PHPT were included. The median age was 61 years (IQR: 53–68), and 73% were female. Regarding comorbidities, hypertension was present in 61% of the cohort, dyslipidemia in 45%, and type 2 diabetes mellitus in 21%. Chronic kidney disease was documented in 10% of patients, while 16% had cardiovascular disease and 6% had a history of pulmonary disease. Thyroid disorders were reported in 29% of cases. In terms of nutritional status, 36% had normal BMI, 31% were overweight, and 33% were classified as obese. Most patients were classified as ASA II (62%) or ASA III (30%). Mean preoperative serum calcium was 11.15 ± 0.93 mg/dL and mean preoperative PTH level was 199.7 ± 188.8 pg/mL (median 144.5 pg/mL).

PHPT was hypercalcemic in 95% of patients, while 5% had normocalcemic PHPT. Symptoms associated with PHPT were reported in 72% of patients, most frequently bone pain (60%), renal colic (27%) and nonspecific symptoms such as fatigue or cognitive complaints (17%). Bone mineral density assessment showed osteopenia in 33% and osteoporosis in 26% of cases.

### Intraoperative and postoperative outcomes

The most commonly performed surgical approach was selective parathyroidectomy (74%), followed by bilateral exploration (21%) and unilateral exploration (5%). Intraoperative parathyroid hormone monitoring was used in all patients and demonstrated a > 50% drop in PTH levels in 96% of cases, meeting the Miami criterion for successful excision. Quantitatively, intraoperative PTH decreased from a mean baseline of 176.5–52.4 pg/mL at 10 min post-excision, corresponding to an average reduction of approximately 70%. Among the four patients who did not reach this ≥ 50% decrease threshold, biochemical cure was still observed in part of them on long-term follow-up, suggesting that failure to meet the Miami criterion did not necessarily translate into persistent disease in this cohort.

Only one patient experienced significant intraoperative bleeding, and no cases required intraoperative conversion or additional unplanned procedures. The number of resected glands was one in 86% of patients, two in 8%, and three in 6%. The most frequently excised glands were the right inferior (26%), left inferior (23%), left superior (21%), and right superior (15%). Histopathological analysis confirmed a single parathyroid adenoma in 87% of cases. Hyperplasia was present in 11% of patients, which corresponds to multiglandular disease (MGD) according to the WHO 2022 classification, and a mixed pattern was observed in 2%. No cases of parathyroid carcinoma were identified.

A total of 93% of patients had a hospital stay of less than 24 h, and there were no readmissions or 90-day mortality. Furthermore, 93% of patients achieved recovery from the disease six months after surgery.

### Textbook outcome achievement and reasons for failure

**Fig. 1:**
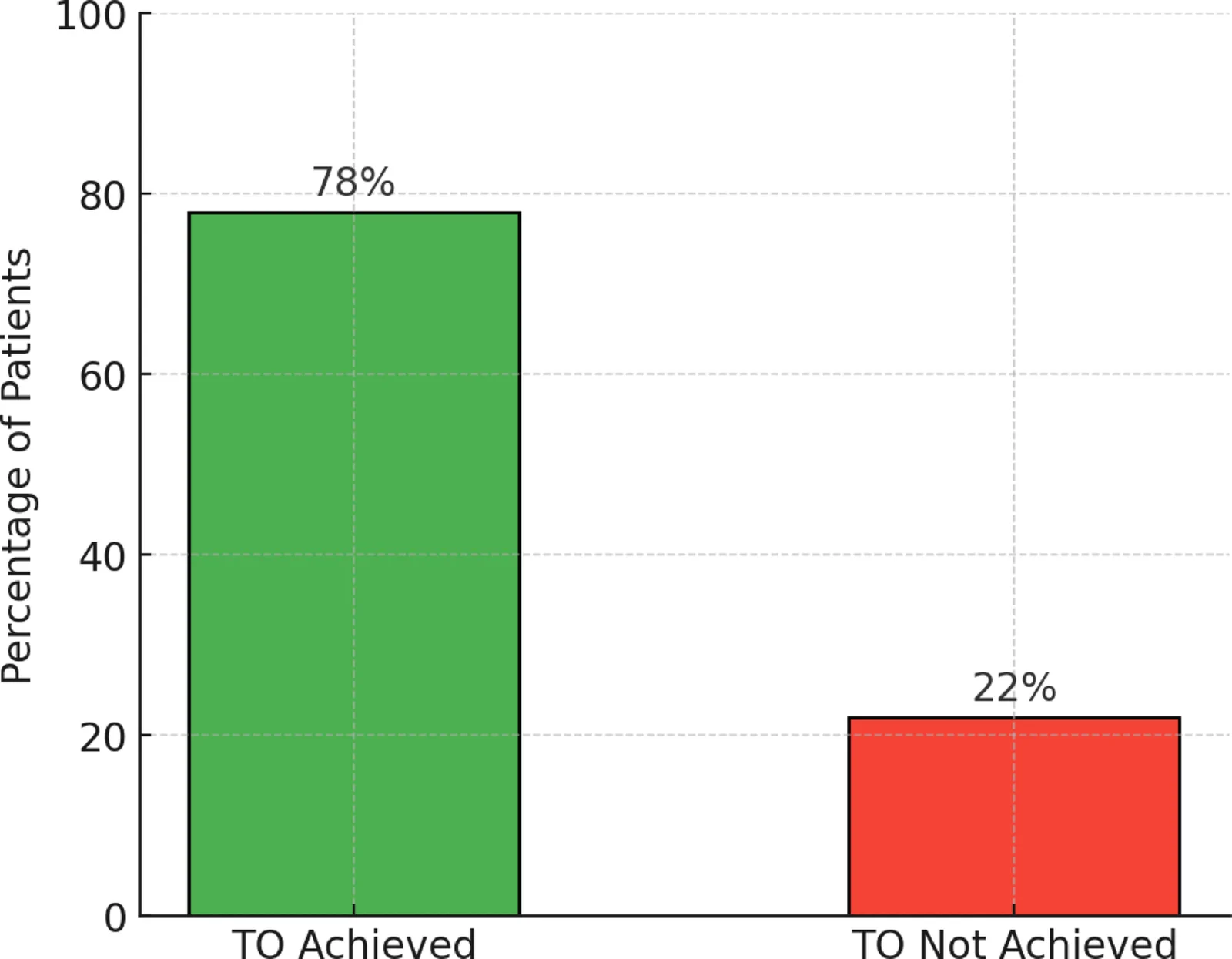
Rate of Textbook Outcome (TO) achievement after parathyroidectomy in the entire cohort (n = 100)

TO was achieved in 78% of the patients (n = 78), meaning that these patients met all the predefined criteria reflecting an optimal postoperative course (Fig. 1). The TO rate remained similar when restricting the analysis to hypercalcemic PHPT patients (73/95, 76.8%).

**Fig. 2:**
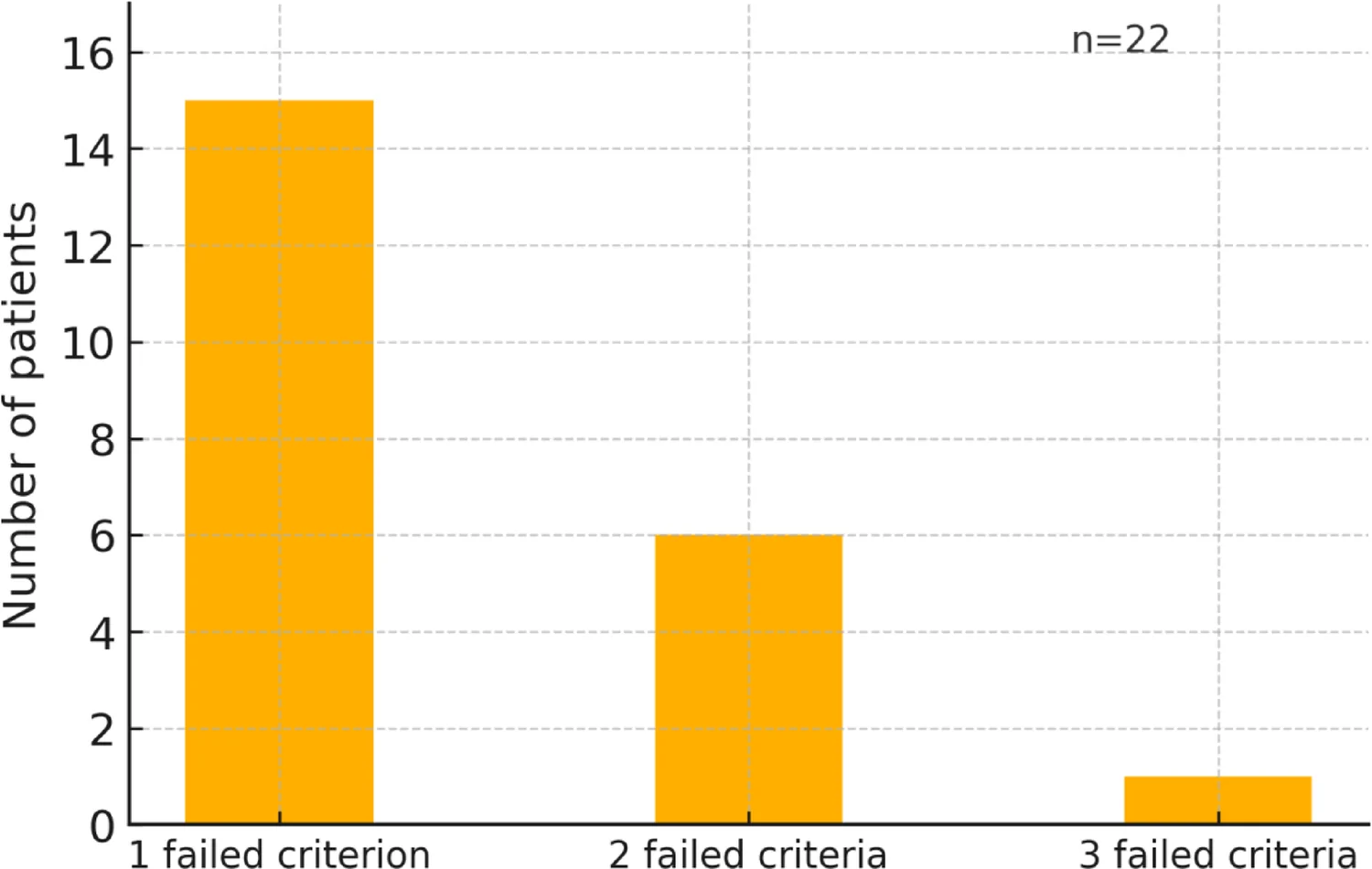
Distribution of the 22 patients who did not achieve Textbook Outcome, according to the number of unmet criteria. Percentages are calculated relative to this subgroup (n = 22), corresponding to 22% of the total cohort (n = 100)

Most patients who failed to achieve TO (n = 22) did so due to the failure of only one criterion (68.2%, n = 15), while 27.3% (n = 6) failed two, and a single patient (4.5%) failed three criteria (Fig. 2).

Among the 22 patients who did not achieve TO, the most frequent reasons for failure were hospital stay longer than 24 h (7 patients, 31.8% of the non-TO group; 7% of the total cohort) and failure to achieve biochemical cure at six months (7 patients, 31.8%; 7% of the total cohort). In most cases, prolonged hospitalization was related to clinical observation needs, particularly transient hypocalcemia symptoms or delayed pain control and mobilization. In patients with relevant comorbidities, extended monitoring was occasionally required. Surgical wound complications were observed in 6 patients (27.3%; 6% of the total cohort), while symptomatic hypocalcemia within the first 24 h occurred in 5 patients (22.7%; 5% of the total cohort). Recurrence within one year was also documented in 5 patients (22.7%; 5% of the total cohort). Postoperative dysphonia was the least frequent cause, affecting 2 patients (9.1%; 2% of the total cohort) (Table 1). Both cases were transient and resolved spontaneously; no permanent recurrent laryngeal nerve injuries were documented. Serum calcium averaged 9.11 ± 1.01 mg/dL at 24 h and 9.61 ± 0.46 mg/dL at 6 months, while parathyroid hormone levels normalized at 12 months, with a mean value of 59.6 ± 36.9 pg/mL.

**Table 1:** Failure components in patients not meeting Textbook Outcome criteria

Criteria Not Met	Number of patients	Percentage*
Hospital stay > 24 h	7	31.8% [7%]
Failure to achieve biochemical cure at 6 months	7	31.8% [7%]
Surgical wound complication	6	27.3% [6%]
Symptomatic hypocalcemia within 24 h	5	22.7% [5%]
Recurrence within one year	5	22.7% [5%]
Postoperative dysphonia	2	9.1% [2%]

^*^Percentages are primarily calculated relative to the 22 patients without TO. Values in brackets indicate the proportion relative to the entire cohort (n = 100)

### Univariate analysis of predictive factors for TO

A univariate analysis was conducted to identify patient variables associated with achieving a TO (Table 2). Among the preoperative characteristics, the absence of dyslipidemia was significantly associated with TO achievement (*p* = 0.016). Intraoperatively, the resection of a single parathyroid gland was also significantly linked to TO (*p* = 0.022).

**Table 2:** Univariate analysis of clinical and intraoperative variables stratified by textbook outcome (TO) achievement

Variable	TO achieved (n = 78)	TO not achieved (n = 22)	OR (95% CI) (TO failure)	*p*-value
Dyslipidemia	30 (38.5%)	15 (68.2%)	3.43 (1.25–9.38)	**0.016**
Number of glands resected				**0.022**
1 gland (single-gland excision)	70 (89.7%)	16 (72.7%)	–	
2 glands	6 (7.7%)	2 (9.1%)	1.46 (0.27–7.90)	
3 glands	2 (2.6%)	4 (18.2%)	8.75 (1.47–52.00)	
Histopathology				**0.026**
Adenoma	69 (88.5%)	18 (81.8%)	–	
Hyperplasia	9 (11.5%)	2 (9.1%)	0.85 (0.17–4.29)	
Mixed/other	0 (0.0%)	2 (9.1%)	18.78 (0.86–408.46)	
Hypertension	44 (56.4%)	17 (77.3%)	2.63 (0.88–7.84)	0.088
Body mass index category				0.13
BMI < 25	23 (29.5%)	8 (36.4%)	–	
BMI 25–29.9	23 (29.5%)	10 (45.5%)	1.25 (0.42–3.74)	
BMI ≥ 30	32 (41.0%)	4 (18.2%)	0.36 (0.10–1.34)	
IOPTH significant drop (Miami criterion met)	76 (97.4%)	20 (90.9%)	0.26 (0.03–1.99)	0.209
Age (years)	59.81 ± 11.91	62.50 ± 10.94	–	0.324
Preoperative calcium (mg/dL)	11.04 ± 0.75	11.50 ± 1.36	–	0.38
Cervical exploration type				0.39
Focused	60 (76.9%)	14 (63.6%)	–	
Unilateral	3 (3.8%)	2 (9.1%)	2.86 (0.44–18.75)	
Bilateral	15 (19.2%)	6 (27.3%)	1.71 (0.56–5.21)	
Diabetes mellitus	15 (19.2%)	6 (27.3%)	1.57 (0.53–4.70)	0.393
Chronic anticoagulant therapy	6 (7.7%)	3 (13.6%)	1.89 (0.43–8.28)	0.408
ASA classification				0.415
ASA I	6 (7.7%)	0 (0.0%)	0.24 (0.01–4.43)	
ASA II	47 (60.3%)	15 (68.2%)	–	
ASA III	24 (30.8%)	6 (27.3%)	0.78 (0.27–2.28)	
ASA IV	1 (1.3%)	1 (4.5%)	3.13 (0.18–53.21)	
Sex (male)	23 (29.5%)	4 (18.2%)	0.53 (0.16–1.74)	0.416
Associated thyroid disease	21 (26.9%)	8 (36.4%)	1.55 (0.57–4.23)	0.43
Preoperative PTH (pg/mL)	192.30 ± 145.98	226.09 ± 298.22	–	0.54
Hypercalcemic PHPT	73 (93.6%)	22 (100.0%)	3.37 (0.18–63.28)	0.583
Major comorbidities (main diagnosis)				0.711
Cardiovascular disease	13 (16.7%)	3 (13.6%)	0.97 (0.23–3.98)	
Pulmonary disease	4 (5.1%)	2 (9.1%)	2.09 (0.34–12.91)	
Cancer history	7 (9.0%)	4 (18.2%)	2.39 (0.59–9.63)	
Chronic kidney disease	8 (10.3%)	2 (9.1%)	1.05 (0.19–5.63)	
None	46 (59.0%)	11 (50.0%)	–	
Intraoperative bleeding	1 (1.3%)	0 (0.0%)	1.15 (0.05–29.17)	1.0

Bold type indicates statistical signifi cance (*p* 0.05)

Other variables such as patient age, sex, BMI, hypertension, diabetes mellitus, ASA classification, associated thyroid disease, presence of nonspecific symptoms, surgical approach, intraoperative bleeding, and PTH drop according to Miami criteria did not show a statistically significant association with TO in this cohort.

## Discussion

In our cohort of patients undergoing parathyroidectomy for PHPT, the overall achievement of a TO reached 78%, a figure that reflects a highly favorable postoperative course in the majority of cases. Although no previous studies have applied the TO concept specifically to this pathology, our results are consistent with those reported in other areas of endocrine surgery. Costa Navarro et al. documented a TO rate of 62.7% in thyroid surgery performed within an endocrine surgical unit [19]. A recent multicenter European study by Nomine-Criqui et al. reported a 52% TO rate in the specific setting of reoperations for persistent or recurrent PHPT, highlighting the higher morbidity and complexity associated with reinterventions. Taken together, these studies support the applicability of TO as a meaningful composite quality metric across different domains of endocrine surgery, while underscoring the importance of tailoring TO definitions to the specific disease setting and surgical complexity. In contrast, our study evaluates TO in primary sporadic PHPT and applies a TO composite adapted to first-time surgical treatment, incorporating early biochemical cure and perioperative metrics relevant to this clinical context [8]. The slightly higher rate observed in our series may be explained by the fact that all included patients underwent primary surgery for sporadic disease in a high-volume center, with well-established protocols and endocrine surgery expertise. Importantly, this performance was consistent in a sensitivity analysis restricted to hypercalcemic PHPT patients, supporting that TO achievement in our cohort was not driven by the small subset of normocalcemic PHPT cases.

Beyond endocrine surgery, the TO has gained recognition as a global performance indicator in various surgical fields. In colorectal cancer surgery, where this concept was first introduced by Kolfschoten et al. in 2013, the TO rate was 49% [6], while more recent studies in hepatobiliary and pancreatic surgery report rates ranging from 50 to 75%, depending on the definition used and patient complexity [9, 20, 21]. These variations underline the importance of adapting TO definitions to the specific context of each disease and surgical procedure, as done in our study. The relatively high TO rate observed in our cohort likely reflects the reproducibility of parathyroidectomy in well-selected patients, as well as the low baseline morbidity profile of the procedure.

The most frequent reason for TO failure in our cohort was hospital stay longer than 24 h (18.2%), followed by biochemical failure at six months (18.2%) and symptomatic hypocalcemia within the first 24 h postoperatively (9.1%). Most biochemical failures occurred in patients with multiglandular disease, corresponding to cases of histologically confirmed hyperplasia. Importantly, intraoperative PTH still met Miami-based decrease thresholds in most of these cases, suggesting that persistence may be explained more by multigland physiology rather than insufficient intraoperative biochemical monitoring. In defining the TO, asymptomatic postoperative hypocalcemia—although often transient—was not included among the criteria, as it does not impact the patient’s clinical course. Instead, only symptomatic hypocalcemia—manifesting as paresthesias or tetany—was considered, due to its clearer clinical relevance and need for therapeutic intervention. This decision aimed to avoid overestimating failure rates based on mild, self-limited biochemical changes that do not compromise long-term outcomes.

This approach aligns with the concern raised in previous studies about the potential over-penalization of procedures due to the inclusion of transient, non-significant events as failure criteria [5]. Indeed, in our series, only two patients (9.1% of the non-TO group) presented early symptomatic hypocalcemia requiring calcium supplementation, and both cases resolved completely in the follow-up. Surgical wound complications occurred in 6 patients (6% of the entire cohort). These included minor events such as seroma, superficial infection, or partial wound dehiscence. All cases were self-limited, managed conservatively, and did not result in reoperation or long-term sequelae. This reinforces the idea that composite indicators like TO must balance sensitivity and clinical relevance, and supports the growing trend to tailor TO definitions to each surgical procedure, especially in low-morbidity contexts [7, 22].

It should be noted that the definition of TO applied in this study was deliberately ambitious, encompassing nine simultaneous criteria. While this comprehensive approach ensured a stringent evaluation of outcomes, it may also have reduced the overall rate of achievement and limited clinical applicability. The challenge of balancing sensitivity with clinical relevance has been highlighted in previous literature on composite outcomes, and further research is warranted to refine and validate TO definitions that are better adapted to endocrine surgery.

Future studies with larger samples are warranted to further explore variables that may specifically influence the main causes of TO failure, such as prolonged hospital stay, symptomatic hypocalcemia in the immediate postoperative period, and failure to achieve biochemical cure. Identifying predictors of these outcomes could provide valuable information for optimizing perioperative management and developing targeted strategies to reduce TO failure rates.

The univariate analysis identified several patient variables significantly associated with the achievement of TO. Among them, the absence of dyslipidemia was notably linked to better outcomes. This finding may reflect the influence of underlying metabolic status on surgical recovery and global postoperative evolution. Although dyslipidemia is not typically considered a direct surgical risk factor, previous studies have shown its association with systemic inflammation, vascular dysfunction, and subclinical organ damage [23], which may complicate perioperative management and recovery. Observational studies have suggested that parathyroidectomy may lead to partial improvement in lipid profiles, particularly in patients with mild PHPT, supporting the notion that dyslipidemia and parathyroid dysfunction may be interrelated conditions that affect patient outcomes [24, 25].

The association between the absence of dyslipidemia and TO achievement should be interpreted with caution. Given the small sample size and the exploratory nature of the analysis, this finding is better regarded as hypothesis-generating rather than conclusive. Nevertheless, it raises the possibility that underlying metabolic status may influence postoperative recovery and warrants further investigation in larger cohorts.

From an intraoperative perspective, the resection of a single parathyroid gland was also significantly associated with TO achievement. This association is consistent with previous reports suggesting that patients with uniglandular disease tend to follow more favorable surgical courses, often requiring shorter operative times, less tissue manipulation, and lower risks of complications. Moreover, the correlation between TO and the histopathological finding of a single adenoma reinforces this observation, as solitary adenomas are the most frequent and technically accessible cause of PHPT [26]. It is worth noting that other traditionally relevant preoperative variables—such as age, sex, ASA classification, BMI, hypertension, diabetes, or the presence of thyroid disorders—did not show a significant association with TO in this cohort. This may reflect the relatively homogeneous low-risk profile of patients undergoing parathyroidectomy in modern endocrine surgery settings, where surgical outcomes are generally favorable and complication rates are low.

The incorporation of TO as a composite outcome measure in endocrine surgery offers significant advantages in both clinical decision-making and institutional quality benchmarking. Unlike isolated indicators, TO captures a more comprehensive and patient-centered snapshot of surgical success. This aligns with a growing interest in surgical quality metrics that reflect not only technical efficacy but also safety, efficiency, and patient recovery, as recent studies have highlighted additional postoperative benefits, such as objectively measurable cognitive improvements following parathyroidectomy for PHPT [27].

In other surgical fields, such as colorectal, hepatopancreatobiliary, and oncologic surgery, TO has been used to compare institutional performance, establish quality standards, and stratify surgical risk [6, 20, 28, 29]. Its application has allowed for the identification of high-performing centers, as well as areas for targeted improvement in perioperative care and surgical pathways [21, 22]. In some studies, TO achievement has even shown a positive correlation with long-term oncologic outcomes and survival, underscoring its prognostic value beyond the immediate postoperative period [9]. Although endocrine surgery has traditionally reported low morbidity and high cure rates, the introduction of TO may reveal differences in outcomes across units or procedures. In low-risk surgeries such as parathyroidectomy, composite indicators like TO offer discriminatory power. Moreover, they may serve as internal audit tools to refine clinical protocols, enhance perioperative coordination, and promote shared goals among multidisciplinary teams.

Beyond feasibility, the implementation of TO in parathyroid surgery may have practical implications. First, TO could serve to identify candidates for outpatient or short-stay surgery, supporting more efficient perioperative pathways. Second, TO achievement rates may be used for benchmarking among surgeons or institutions, fostering quality improvement initiatives. Finally, by integrating multiple domains of safety and efficacy, TO provides a comprehensive framework that can guide institutional audits and patient-centered outcome evaluation in endocrine surgery.

## Limitations of the study

This study has several limitations inherent to its retrospective and single-center design. The analysis may be subject to selection bias and limited external validity. Additionally, the relatively small sample size, although adequate for exploratory analysis, may reduce the statistical power to detect associations in less frequent variables. Only univariate associations were assessed, and no multivariate model was constructed given the limited number of events. Therefore, the identified predictors should be interpreted as exploratory and hypothesis-generating rather than definitive. Future studies including larger and multicenter cohorts will be required to enable robust multivariate analyses and to identify independent predictors of TO achievement.

Hospital stay was the most frequent reason for TO failure. This criterion, however, may be influenced not only by clinical factors but also by institutional policies or logistical considerations, potentially limiting its discriminatory power as a quality indicator. The absence of follow-up beyond one year restricts the evaluation of late recurrence or delayed complications. Although this interval allowed reliable assessment of biochemical cure and early persistence, it may underestimate recurrences that manifest later. Longer follow-up will therefore be necessary to validate the durability of TO in parathyroid surgery. Lastly, while the TO definition was carefully adapted to the context of parathyroid surgery, there is currently no universally validated standard for TO in this specific field, which may affect comparability with future studies.

## Conclusions

The implementation of TO as a composite metric in parathyroid surgery for PHPT is feasible and informative, with 78% of patients meeting TO criteria. Analysis of failures identified prolonged hospital stay and biochemical failure at six months as the most frequent reasons for non-achievement. Absence of dyslipidemia, resection of a single gland, and histopathological confirmation of a single adenoma were associated with higher rates of TO, although these findings should be regarded as exploratory. By integrating key clinical and surgical outcomes, TO provides a comprehensive benchmark that complements traditional outcome measures and holds promise as a tool for quality improvement and benchmarking in endocrine surgery.

## Data Availability

The datasets generated and/or analyzed during the current study are not publicly available due to privacy and ethical restrictions but are available from the corresponding author upon reasonable request.
